# Novel lipid-coated mesoporous silica nanoparticles loaded with thymoquinone formulation to increase its bioavailability in the brain and organs of Wistar rats

**DOI:** 10.1186/s40360-022-00616-z

**Published:** 2022-09-26

**Authors:** Heba M. Fahmy, Mostafa M. Ahmed, Ayman S. Mohamed, Engy Shams-Eldin, Taiseer M. Abd El-Daim, Amena S. El-Feky, Amira B. Mustafa, Mai W. Abd Alrahman, Faten F. Mohammed, Mohamed M. Fathy

**Affiliations:** 1 Biophysics Department, Faculty of Science, Cairo University, Giza, Egypt; 2 Microbiology Department, Faculty of Science, Cairo University, Giza, Egypt; 3 Zoology Department, Faculty of Science, Cairo University, Giza, Egypt; 4 Special Food and Nutrition Department, Food Technology Research Institute, Agriculture Research Center, 9 Gamma Street, Giza, Cairo, Egypt; 5 School of Biotechnology, Badr University in Cairo, Badr City, Cairo, 11829 Egypt; 6 Pathology Department, Faculty of Veterinary, Cairo University, Giza, Egypt

**Keywords:** Thymoquinone, Mesoporous silica nanocarriers, Lipid bilayer, Biodistribution, Oxidative stress, Brain areas

## Abstract

**Aims:**

The Blood-Brain Barrier (BBB) is a filter for most medications and blocks their passage into the brain. More effective drug delivery strategies are urgently needed to transport medications into the brain. This study investigated the biodistribution of thymoquinone (TQ) and the effect on enzymatic and non-enzymatic oxidative stress indicators in different brain regions, either in free form or incorporated into nanocarriers as mesoporous silica nanoparticles (MSNs). Lipid bilayer-coated MSNs.

**Materials and methods:**

MSNs and LB-MSNs were synthesized and characterized using a transmission electron microscope and dynamic light scattering to determine the particle size and zeta potential. TQ encapsulation efficiency and TQ's release profile from LB-MSNs were also examined. The impact of loading LB-MSNs with TQ-on-TQ delivery to different brain areas was examined using chromatographic measurement. Furthermore, nitric oxide, malondialdehyde (MDA), reduced glutathione, and catalase were evaluated as oxidant and antioxidant stress biomarkers.

**Key findings:**

The LB-MSNs formulation successfully transported TQ to several areas of the brain, liver, and kidney, revealing a considerable increase in TQ delivery in the thalamus (81.74%) compared with that in the free TQ group and a considerable reduction in the cortex (−44%). The LB-MSNs formulation had no significant effect on TQ delivery in the cerebellum, striatum, liver, and kidney.

**Significance:**

TQ was redistributed in different brain areas after being encapsulated in LB-MSNs, indicating that LB-MSNs have the potential to be developed as a drug delivery system for selective clinical application of specific brain regions.

**Conclusions:**

LB-MSNs are capable nanoplatforms that can be used to target medications precisely to specific brain regions

## Background

The Blood-Brain Barrier is supposed to be an obstruction for treating neurodegenerative disorders because it filters the delivery of most Central Nervous System (CNS) active medicines. The BBB prevents the flow of potentially harmful substances to protect the brain [1]. Recently, nanomedicine's new approaches tend to overcome BBB for drug delivery into CNS.

Mesoporous silica is used *in vivo* as an intelligent drug delivery system to treat many neurodegenerative illnesses. Silica is a natural compound used as secure and cost-effective material with few side effects [2]. MSNs are chemically stable compounds; they can be easily synthesized with a large surface area, and diverse pore diameters may be designed to transport various medicines of various sizes [3].

Nanotechnology-synthesizing techniques improve MSNs to be more biocompatible, have better biodistribution, possess controlled drug release regardless of the surrounding environment, and have good drug loading capacity with better cellular uptake and negligible toxicity [4]. Baghirov *et al*. studied MSNs' effect on BBB. Functionalized nanocarrier with PEG–PEI copolymers. This polymer coating improved rod-shaped MSNs absorption by brain cells without damaging the BBB or being toxic [5]. Numerous studies have demonstrated that MSNs effectively target receptors on the endothelial cell membrane of the brain, including transferring (Tf), low-density lipoprotein, lactoferrin (Lf), mannose, folic acid (FA), RGD (arginine–glycine–aspartic acid), and insulin [6]. Song *et al*. functionalized MSNs with Lf, which caused Lf-mediated transcytosis. They covered the nanocarrier's surface with PEG to prevent reticuloendothelial system cleaning. In brain cells, 25 nm nanoparticles had the most efficient transport. Lf-functionalized MSNs that cross the BBB via receptor-mediated transcytosis can transfer drugs to the brain [7].

The active phytochemical component of *Nigella Sativa*, or black cumin, is thymoquinone; it possesses vital characteristics in treating neurodegenerative diseases like multiple sclerosis (MS) for its anti-inflammatory, antioxidant, and immunomodulatory ingredients [8]. It can inhibit the pro-inflammatory cytokines by targeting different signaling pathways to control the pro-inflammatory gene expression and regulate immune response [9]. TQ is also a controller for oxidative stress because it reduces the reactive oxygen species by holding O^-2^, OH^ˉ^, and H_2_O_2_ free radicals [10]. As much as this molecule has been explored in clinical trials, its limited bioavailability and hydrophobicity have kept it from being widely used. Consequently, putting TQ into silica nanostructures improves its bioavailability to a greater extent than free TQ [11]. Shahein *et al.* designed TQ: MSNs with a shell made of two different polymers. The pH was changed to make the drug come out. TQ-MSNs showed high drug release at pH 7.4, while TQ-MSNs with whey protein and gum Arabic shell and TQ-MSNs with chitosan and stearic acid shell released TQ at pH 5.5 and 6.8, respectively. The drug is most effectively released in the tumor's acidic environment. Also, the way these nanoparticles fought cancer was more potent than free TQ and less harmful to cells [12].

Our study aims to evaluate a new formulation of thymoquinone-loaded mesoporous silica nanocarrier with additional lipid coating (liposomes), to enhance better biological/cellular interaction through BBB. It might improve the biocompatibility of the formulation and optimize the drug's effectiveness [13]. This is followed by comparing the biodistribution of thymoquinone and various oxidative and antioxidant stress biomarkers (enzymatic and non-enzymatic) in different brain parts of each form (free TQ or LB-MSNs).

## Materials and methods

### Materials

Thymoquinone (2-isopropyl-5-methyl-1, 4-benzoquinone), cetyltrimethylammonium bromide (CTAB) (99%), 99% ethanol, tetraethyl orthosilicate (TEOS, 99%), dimethyl sulfoxide (DMSO), chloroform and asolectin lipid were all ordered from Sigma–Aldrich (St. Louis, MO, USA). Ammonium hydroxide (NH_4_OH, 28%) was brought from Fluka. Phosphate buffered saline (PBS, pH 7.4) was obtained from Bio Diagnostic Co., Giza, Egypt. 2-ethoxyethanol (C_2_H_5_OCH_2_CH_2_OH, 99%) was received from Merck.

### Methods of Preparation

#### Mesoporous silica nanoparticles

Synthesis of mesoporous nanoparticles based on Fahmy *et al.* [14, 15]. Firstly, a magnetic stirrer was used to prepare an apparent solution by adding 0.5gm of CTAB (cationic surfactant) to 70 ml of deionized water. The solution was mixed with 0.5 ml NH_4_OH and 30 ml 2-ethoxyethanol (co-solvent). Stirring was continued at room temperature for 30 minutes, followed by adding 2.5 ml of TEOS (a source of silicon) and vigorous stirring for 24 hours. The sample was then calcined for six hours at 600°C to eliminate any remaining CTAB. The prepared silica was extracted after centrifuging it for 20 minutes at 6000 rpm and rinsing it with ethyl alcohol and deionized water.

#### Mesoporous silica nanoparticles loaded with thymoquinone

We dissolved thymoquinone in DMSO and saline at a 1: 9 DMSO: saline ratio [10, 11] for drug loading. After that, the TQ solution was slowly introduced to the MSNs sample. (With constant stirring, MSNs are suspended in deionized water at a ratio of 1:1, w/w.)

The suspensions were mixed for 24 hours at room temperature in a water bath at 100 rpm.

#### Mesoporous silica nanoparticles loaded with thymoquinone and coated with a lipid bilayer

With some modifications, LB-MSNs were prepared as per Tu *et al*. [16]. For liposome synthesis, asolectin lipid (approximately 24% saturated, 14% monounsaturated, and 62% polyunsaturated fatty acids) was emulsified in chloroform and stored overnight under slow but steady evaporation by a nitrogen flow (under vacuum) to form a thin lipid film. The lipid film was rehydrated with PBS (2 ml, one mM, pH 7.4), followed by a vortex to generate a hazy lipid suspension. It was sonicated in a water bath at 50°C for 10 minutes. Liposomes were created by the previously mentioned method, which may be preserved at 4°C. To generate the LB-MSNs, 0.5 ml TQ-loaded MSNs were dispersed in 1 mg/ml PBS combined with 0.5 ml of the previously prepared liposomes and shaken at 400 rpm for approximately an hour and a half. LB-MSNs were then isolated through centrifugation at 13,000 rpm for 5 minutes and washed (three times) using PBS. The free TQ concentration in the supernatant was determined using a spectrophotometer calibrated using TQ's calibration curve at wavelength = 253 nm, and the encapsulation efficiency was determined using the calibration curve following equation 1.

1 $$EE\%=\left(\frac{total\kern0.5em drug- freedrug}{total\kern0.5em drug}\right)x100$$

### Physical characterization

#### Transmission electron microscopy (TEM)

The morphological characteristics of the prepared MSNs-TQ and LB-MSNs were investigated by TEM (JEM 1230 electron microscope JEOL, Tokyo, Japan). TEM can provide information about the crystallization of the structure and the nanoparticles' size. Each sample was suspended in deionized water on a carbon-coated copper grid. Before examining the grid, it was allowed to dry for about 5 minutes at room temperature.

#### Particle size (PS) using Dynamic light scattering (DLS) technique

The synthesized formulations' hydrodynamic diameter and size distribution, MSNs-TQ and LB-MSNs, were determined using the Zetasizer (Nano ZS, Malvern Instruments, UK). The sample was analyzed in quartz cuvettes, the temperature was set to 25 °C, and the viscosity and refractive index values used were that of pure water. This instrument utilizes laser diffraction to detect scattered laser light at a 90° angle and ambient temperature for diluted samples. A Polydispersity Index (PDI) is also provided as part of this approach, which provides information on the sample's polydispersity.

#### Zeta *potential*

Zetasizer was also used to determine the surface charge of the prepared samples at ambient temperature (Nano ZS, Malvern Instruments, Malvern, UK). Zetasizer was also used to determine the surface charge of the prepared samples at ambient temperature (Nano ZS, Malvern Instruments, Malvern, UK). The zeta values were obtained by the electrophoretic mobility of nanoparticles moving into an applied electric field.

#### In vitro drug release

A dialysis bag diffusion approach was used to determine the *in vitro* TQ release profile of LB-MSNs. The dialyzer used for the release experiment was conducted using clean dialysis bags with a 12,000 DA molecular weight limit. The release material was immersed in these bags for 24 hours (distilled water). Ten milliliters of LB-MSNs were injected into the dialysis bags. Release media (30 ml) was added to each container after the closed dialysis bags were inserted. The bottles were shaken at 100 revolutions per minute at 37 degrees Celsius. Two ml of the external media was removed from the beaker's center at predefined time intervals and replaced with a new medium (0.5, 1, 1.5, 2, 3, 4, 5, 6...,48 h). The concentrations of liberated TQ were measured using the calibration curve and a UV–Vis Spectrophotometer calibrated to 253 nm. (Jenway UV-6420; Barloworld Scientific, Essex, UK).

### In vivo experiments

#### Animals

Twenty-eight male Wistar rats with an average weight of 110±10 gm were allocated into four random groups (seven rats/group). Animals were kept in fixed housing circumstances (12 h light/dark cycles) and temperature (25 °C) for three days to acclimatize. The Institutional Animal Ethics Committee approved the protocol for the *in vivo* study (CU-I-F-28-21).

#### Experimental design

The four groups received oral dose day by day for 14 days as follows: The first group: the negative control group, received 1:9; DMSO: Saline. The second group: the free TQ group, received a 60 mg/kg free TQ (3). The third group: The MSNs group received a 60 mg/kg MSNs dose. The fourth group: the LB-MSNs group, received a dose of 60 mg/kg MSNs loaded with TQ and coated with lipids. On day 14, rats were decapitated, and rat brains, liver, and kidneys were dissected for histopathological examination and oxidative stress measurements. The duration time of our experiment and the TQ doses were determined following Sheikhbahaei et al. (2016). 's work [17].

#### Handling of tissue samples

On the 14^th^ day of the experiment, all the experimental groups were sacrificed by decapitation. The brains of each animal were taken away after they were removed and put in an ice-cold Petri dish. The brain was sectioned into different regions (hippocampus, cortex, hypothalamus, thalamus, medulla, midbrain, cerebellum, and striatum). Each brain region was weighed and frozen until used in further measurements. Heidolph DIAX 900 was used to homogenize the brain sections weighed in 4 ml of ice-cold PBS (50 mM, pH 7.4). Then, for 15 minutes, the homogenates were separated using a high-speed cooling centrifuge. The centrifuge was set to 8000 RPM and 4°C (VS-18000 M small size, high speed, refrigerated centrifuge, Korea). The pure supernatant was collected and stored at -20°C for further analysis.

#### The distribution of TQ in various areas of the brain

TQ was chromatographically identified at room temperature 25 °C. The mobile phase was 50: 30: **20 v/v/v methanol, acetonitrile, and potassium dihydrogen phosphate buffer (20 mM, pH 4.5), isocratically injected at a flow rate of 1 ml/min using the HPLC apparatus's C18 column (25 cm4.6 mm) (YL 9100 HPLC, USA).

#### Oxidative biomarkers

##### Levels of Nitric oxide (NO)

The nitric oxide assay was performed according to the Griess reaction method using biodiagnostic kit No. 25 33, Egypt. The nitrite was transformed into a deep purple azo compound when Griess reagent was added to the tissue supernatant. Absorption was measured using a spectrophotometer at 450 nm (Jenway UV-6420; Barloworld Scientific, Essex, UK). NO levels were quantified as nitrite using the Griess reagent, according to Moshage et al. [18].

##### Lipid peroxidation (MDA) levels

MDA was determined as a marker of lipid peroxidation using a method developed by Begona Ruiz-Larrea et al. [19], which entailed detecting the thiobarbituric acid-reactive molecules. In this approach, the reactive compounds react with the thiobarbituric acid to form a pink complex. A spectrophotometer was used to measure the absorbance of the generated compound at 532 nm (Jenway UV-6420; Barloworld Scientific, Essex, UK).

##### Reduced glutathione (GSH) levels

According to Ellman's method, the determination of Reduced Glutathione concentration (GSH) required using Biodiagnostic kit No. GR 25 11, Egypt. Ellman's method measured reduced glutathione levels, as the sulfhydryl group of GST lowers Ellman's reagent and produces 2-Nitro-smercaptobenzoic acid [20]. The yellow-colored nitro-mercaptobenzoic acid was detected at 412 nm using a spectrophotometer (Jenway UV-6420; Barloworld Scientific, Essex, UK).

##### Catalase levels

The catalase activity was determined according to Aebi’s protocol (spectrophotometry) using Biodiagnostic Kit No. CA 25 1, Egypt [21]. Catalase responds with a specified amount of hydrogen peroxide, and the catalase inhibitor stops the reaction after 1 minute. The residual hydrogen peroxide combines with 3, 5-dichloro-2-hydroxybenzene sulfonic acid (DHBS), and 4-aminophenazone to generate a chromophore with an intensity of color in inverse proportion to the sample's catalase activity in the presence of peroxidase. The chromophore's absorbance at 510 nm is measured using a spectrophotometer (Jenway UV-6420; Barloworld Scientific, Essex, UK).

#### Histopathological assessment

Each group's brain, liver, and kidney were fixed in a formalin buffered solution (10%) for twenty-four hours. Fixed specimens were rinsed with tap water before dehydrating with methyl, ethyl, and absolute dilutions. Specimens were cleaned in xylene and embedded in paraffin wax for 24 hours in a 56°C hot air oven. Microtome blocks of Paraffin bees wax tissue were sectioned at 8 microns on a slide, collected on glass slides, and removed paraffin stained with hematoxylin and eosin (H & E) stains. A light microscope was used to examine the slides (Zeiss, Germany).

### Statistical analysis

We used The Statistical Package for Social Sciences (SPSS) software (version 19) to analyze the data. The mean and standard error of the results were calculated. The Analysis of Variance (ANOVA) test examined the results. For *p*-value, *p* ≤ 0.05, differences were considered significant. The percentage difference, which reflects the percent change in value compared to the control, was also assessed.

$$\% Difference\left(\%D\right)=\left(\frac{treated\kern0.5em value- control\kern0.5em value}{control\kern0.5em value}\right)\times \kern0.5em 100\%$$

## Results

### Characterization of the nanoformulations

**Fig. 1:**
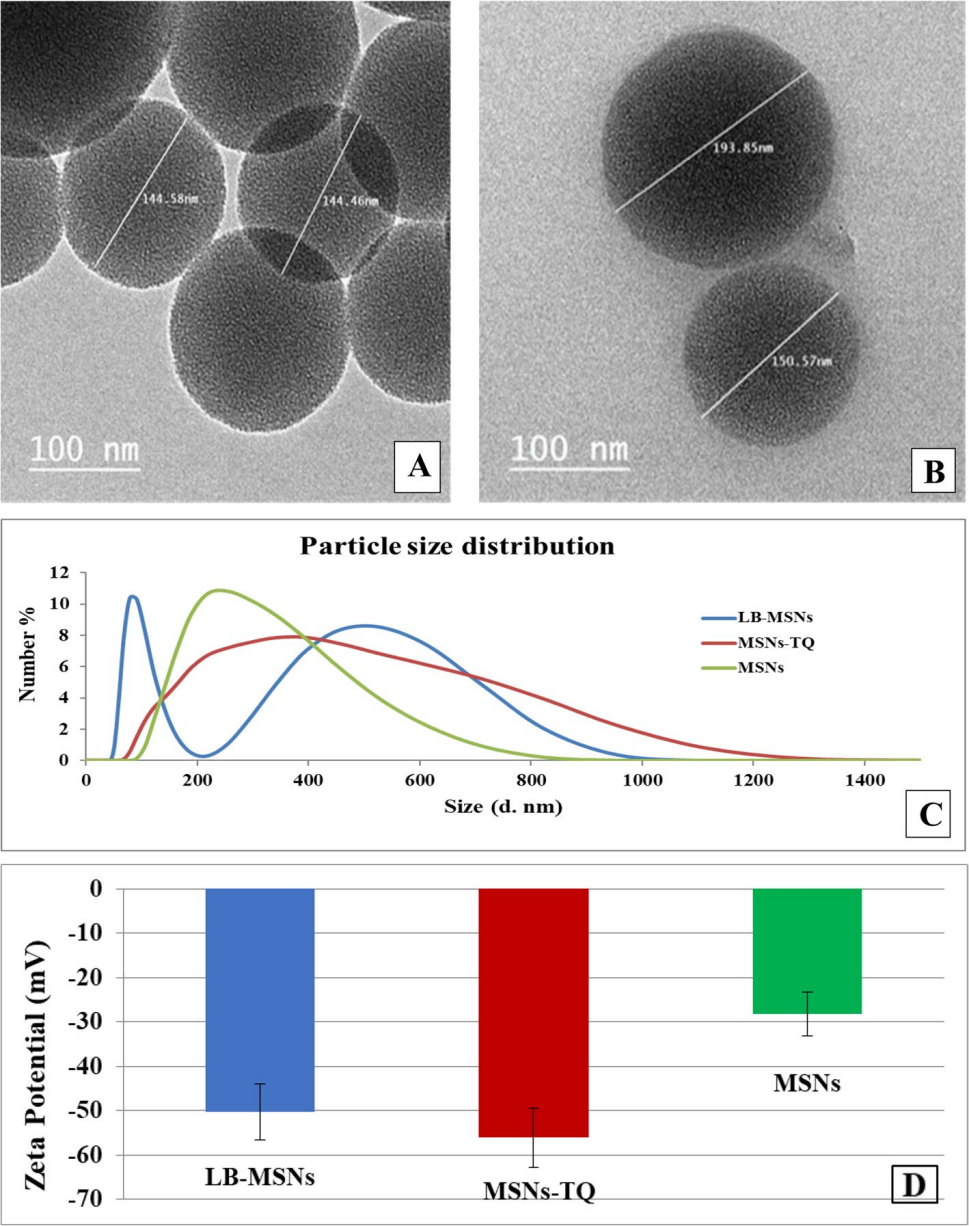
**A** Mesoporous silica nanoparticles (MSNs) TEM micrograph. **B** lipid bilayer-coated mesoporous silica nanoparticles (LB-MSNs) TEM micrograph. **C** Particle size distribution of MSNs, MSNs-TQ, and LB-MSNs. **D** Zeta potential distribution of MSNs, MSNs-TQ, and LB-MSNs; each result represents mean ± standard error (*n* = 3)

A transmission electron microscope (TEM) showed the spherical morphology of MSNs and LB-MSNs. As seen in Fig. 1(A), micrographs show that the MSNs had a small aggregation with a similar pore alignment and were shaped with a mean diameter of 144.52 ± 0.06 nm. Conversely, the prepared LB-MSNs show a spherical shape with a larger mean diameter of 171.21 ± 14.59 nm, whereas the lipid layer thickness was approximately 5.53 nm (Fig. 1B).

As demonstrated in Fig. 1(C), DLS assessment results reveal that the mean hydrodynamic diameters of MSNs, MSNs-TQ, and LB-MSNs are 291.9 ± 64.3, 383 ± 113.5, and 504 ± 73.6 nm, respectively. Furthermore, the polydispersity index (PDI) values are 0.234, 0.251, and 0.474 for MSNs, MSNs-TQ, and LB-MSNs, respectively, which reveal a homogenous distribution for the formulations. For LB-MSNs, it was hypothesized that the development of empty smaller liposomes, smaller in size than MSNs, was responsible for the highest PDI value (0.474) observed.

Zeta potential, which indicates the external surface particle charge, showed that the net charge of the developed MSNs was negative, with an average value of −28.2 ± 2.48 mV. After loading with TQ, the net charge increased to −56.1 ± 6.74 mV. Simultaneously, coating with lipid bilayers decreased the surface zeta potential charge to −50.2 ± 6.35 mV (Fig. 1D). This result (a relatively high zeta potential value) indicates the high stability of the prepared nanocarriers and the absence of aggregation in the samples. The high encapsulation efficiency of MSNs-TQ (93% ± 2.5%) is attributable to the large surface area of MSNs pores, which could receive large TQ quantities.

### In vitro release experiment

**Fig. 2:**
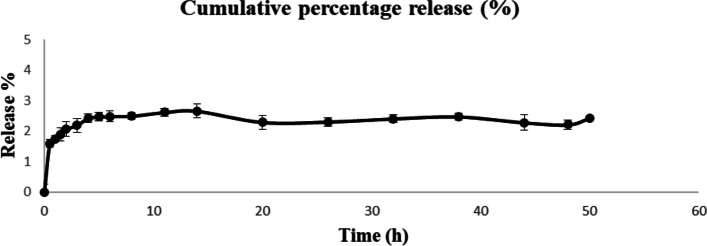
*In vitro* release profile of free TQ form LB-MSNs, each result represents mean ± standard error of the mean (*n* = 3)

The rapid discharge of TQ from LB-MSNs in vitro during the first five h could be attributable to the discharge of TQ from the surface of MSNs. The subsequent gradual release was most likely due to TQ being released through the internal pores of MSNs (Fig. 2). Owing to the possible attraction of Silica in MSNs and oxygen in TQ molecules, the discharge of TQ from the inner pores was hindered, and controlled [22].

### In vivo experiments

#### Distribution of TQ in different brain areas

TQ biodistribution in the distinct brain, liver and kidney areas for the third (TQ group that administered free TQ) and fourth groups (LB-MSNs that administered LB-MSNs −60 mg of TQ/kg to 60 mg of MSNs/kg) is presented in Table 1. Based on the results, TQ loading to LB-MSNs improved the drug's effectiveness, significantly directing it to the thalamus alone (81.74%). However, compared with the free TQ group, the hippocampus, hypothalamus, medulla, and midbrain were enhanced by 399.25%, 251.60%, 76.37%, and 57.14%, respectively. Conversely, TQ encapsulation to LB-MSNs significantly depleted its targeting only to the cortex (44%). TQ biodistribution in the cerebellum, striatum, liver, and kidney was reduced by 74.13%, 94.90%, 43.35%, and 36.72%, respectively.

**Table 1:** TQ distribution (μg/g tissue) in the different areas of the brain, liver, and kidney in the experimental groups

Areas	TQ	LB-MSNs	Significance	%D
	Mean ± SEM	Mean ± SEM		
**Cerebellum**	0.048 ± 0.018	0.012 ± 0.0003	ns	−74.13%
**Cortex**	0.025 ± 0.004	0.014 ± 0.003	*	−44%
**Hippocampus**	0.002 ± 0.0003	0.013 ± 0.0019	ns	399.25%
**Hypothalamus**	0.01 ± 0.0003	0.034 ± 0.0151	ns	251.60%
**Medulla**	0.006 ± 0.001	0.011 ± 0.002	ns	76.37%
**Midbrain**	0.007 ± 0.001	0.011 ± 0.02	ns	57.14%
**Striatum**	0.013 ± 0.001	0.007 ± 0.001	ns	−94.90%
**Thalamus**	0.004 ± 0.001	0.007 ± 0.001	*	81.74%
**Liver**	0.201 ± 0.024	0.114 ± 0.007	ns	−43.35%
**Kidney**	0.112 ± 0.019	0.071 ± 0.015	ns	−36.72%

%D: percentage difference in comparison to the TQ group.

*TQ* Free thymoquinone (60 mg/kg), *LB-MSNs* Lipid bilayer-coated MSNs (60 mg of TQ/kg to 60 mg of MSNs/kg)

*Significant; ns: nonsignificant

*One-way ANOVA test was used as statistical analysis.

#### Oxidant and antioxidant stress biomarkers evaluation

Male Wistar rats with a mean weight of 110 g were utilized. They were kept in standard housing settings, which included 12-h light/dark cycles and a temperature of 25°C ± 1°C. For 14 days, 28 rats were divided into four groups (seven rats/per group) and administered with the following oral doses: (1) control group: rats were administered saline and dimethyl sulfoxide (DMSO) (10% DMSO: 90% saline); (2) MSNs group: rats were administered MSNs (60 mg/kg); (3) TQ group: rats were administered free TQ (60 mg/kg); and (4) LB-MSNs group: rats were administered LB-MSNs (60 mg TQ/kg to 60 mg MSNs/kg) [13]. Previous toxicity experiments were used to determine the trial duration and doses.

**Fig. 3:**
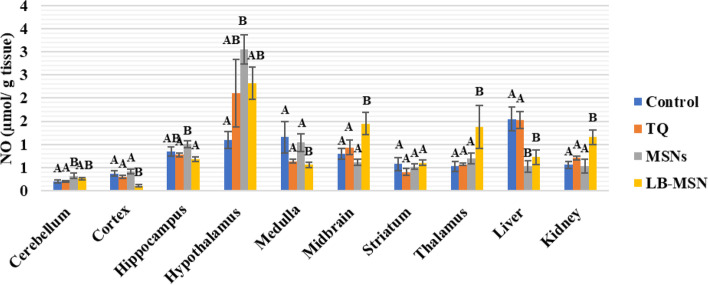
Nitric oxide (NO) levels (μmol/g tissue) in the different areas after treatment with lipid bilayer-coated mesoporous silica nanoparticles loaded with thymoquinone

Rats of the TQ group demonstrated nonsignificant changes in all the tested organs compared with those of the negative control group (Fig. 3). For the MSNs group, NO levels recorded significant increases compared with control values in the following areas: cerebellum and hypothalamus (63.28% and 179.75%, respectively) and a significant decrease in the liver (65.95%); however, no significant changes were recorded in the remaining investigated areas. Regarding control values, NO levels in the rats of the LB-MSNs group showed significant increases in the midbrain, thalamus, and kidney (82.50%, 159.22%, and 107.15%, respectively). However, significant decreases in the cortex, medulla, and liver (−72.89%, −51.61%, and −53.42%, respectively) were also evident.

**Fig. 4:**
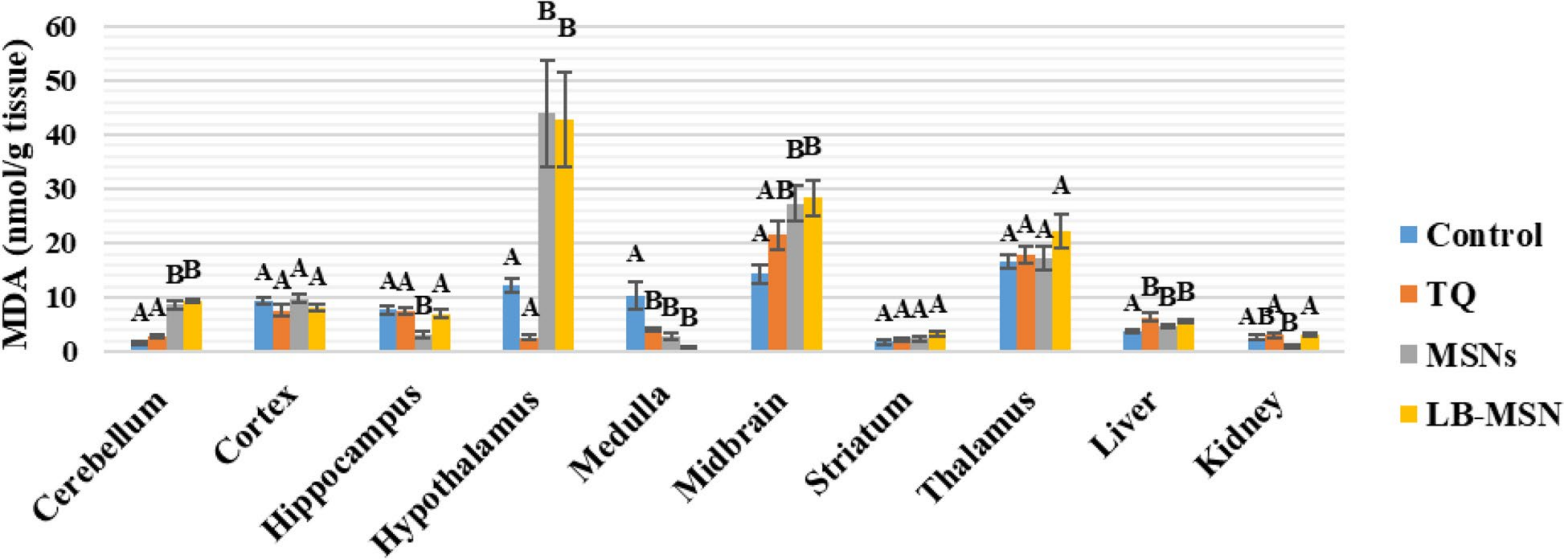
Lipid peroxidation (MDA) levels (nmol/g tissue) in the different areas after treatment with lipid bilayer-coated mesoporous silica nanoparticles loaded with thymoquinone

Fig. 4 reveals that the MDA concentration in the TQ group showed nonsignificant changes in all tested areas except the medulla, which confirmed a significant decrease (60.95%) below the control level. Meanwhile, compared with the negative control group, the MDA concentration in the liver showed a significant increase (67.01%). For the MSNs group, MDA results showed significant increases in the cerebellum, hypothalamus, midbrain, and liver (427.14%, 259.88%, 89.88%, and 22.09%, respectively). Significant decreases in MDA levels were found in the hippocampus, medulla, and kidney (−58.82%, −73.05%, and −58.52%, respectively), as well as nonsignificant alterations in the cortex, striatum, and thalamus (5.45%, 34.86%, and 3.75%, respectively). MDA levels in the LB-MSNs group revealed significant increases in the cerebellum, hypothalamus, midbrain, and liver (477.93%, 249.29%, 96.78%, and 46.70%, respectively) and significant decreases in the medulla only with a percentage difference of 91.02% when compared with control values. Furthermore, in the cortex, hippocampus, striatum, thalamus, and kidney, no significant variations were noted in MDA levels compared with the control group.

**Fig. 5:**
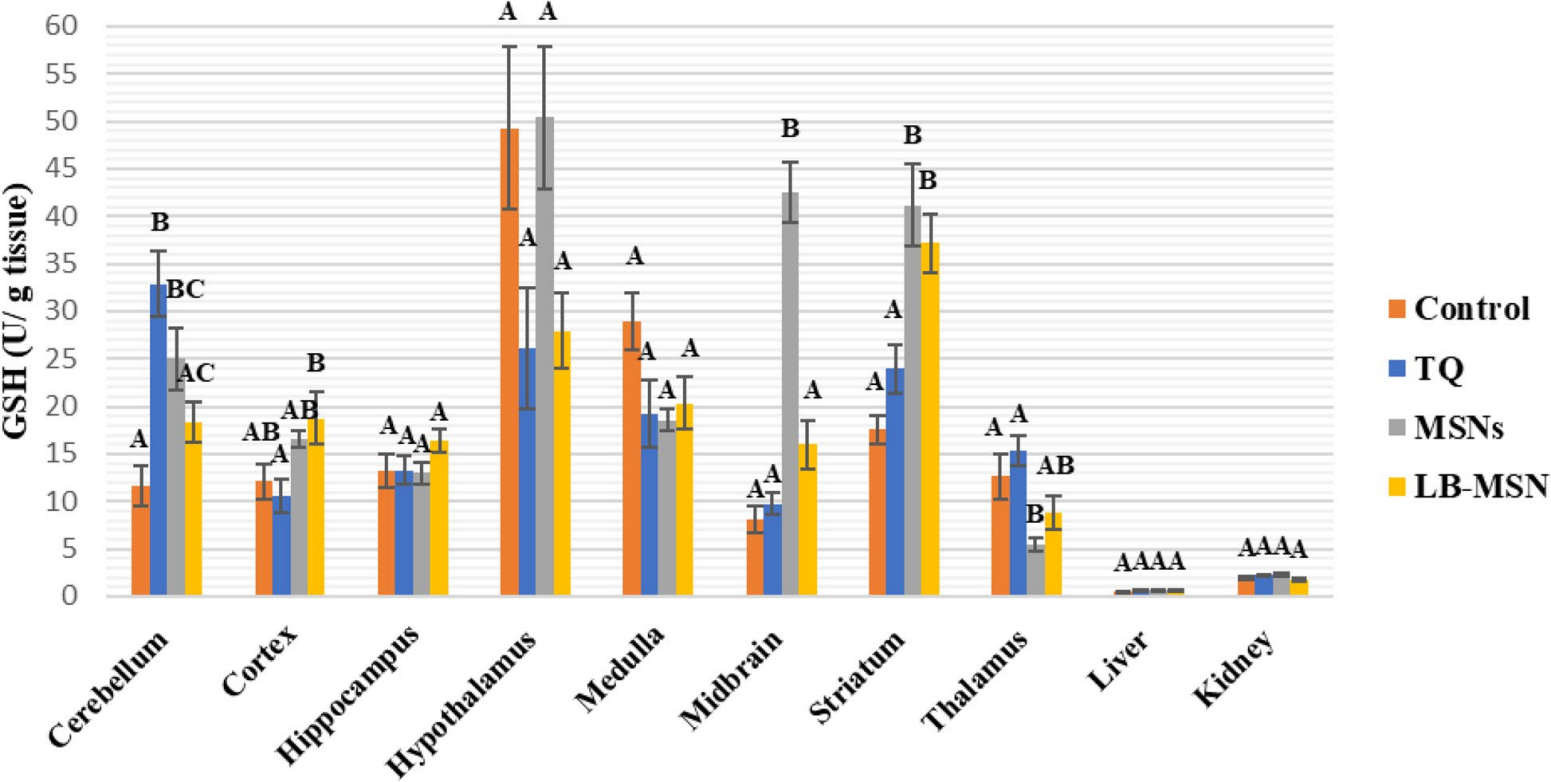
Reduced glutathione (GSH) levels (mmol/g tissue) in the different areas after treatment with lipid bilayer-coated mesoporous silica nanoparticles loaded with thymoquinone

Compared with the negative control group values, the TQ group showed no significant changes in all investigated areas, except for the cerebellum, which showed a considerable increase (181.98%) in glutathione (GSH) levels (Fig. 5). For the MSNs group, GSH levels showed significant increases compared with the control values in the following areas: cerebellum, midbrain, and striatum (114.2%, 423.4%, and 133.7%, respectively), and a significant decrease in the thalamus (56.87%); however, no significant changes were observed in the remaining investigated brain areas and tissues. Regarding control values, the LB-MSNs group demonstrated a significant increase in only the striatum (110.79%).

**Fig. 6:**
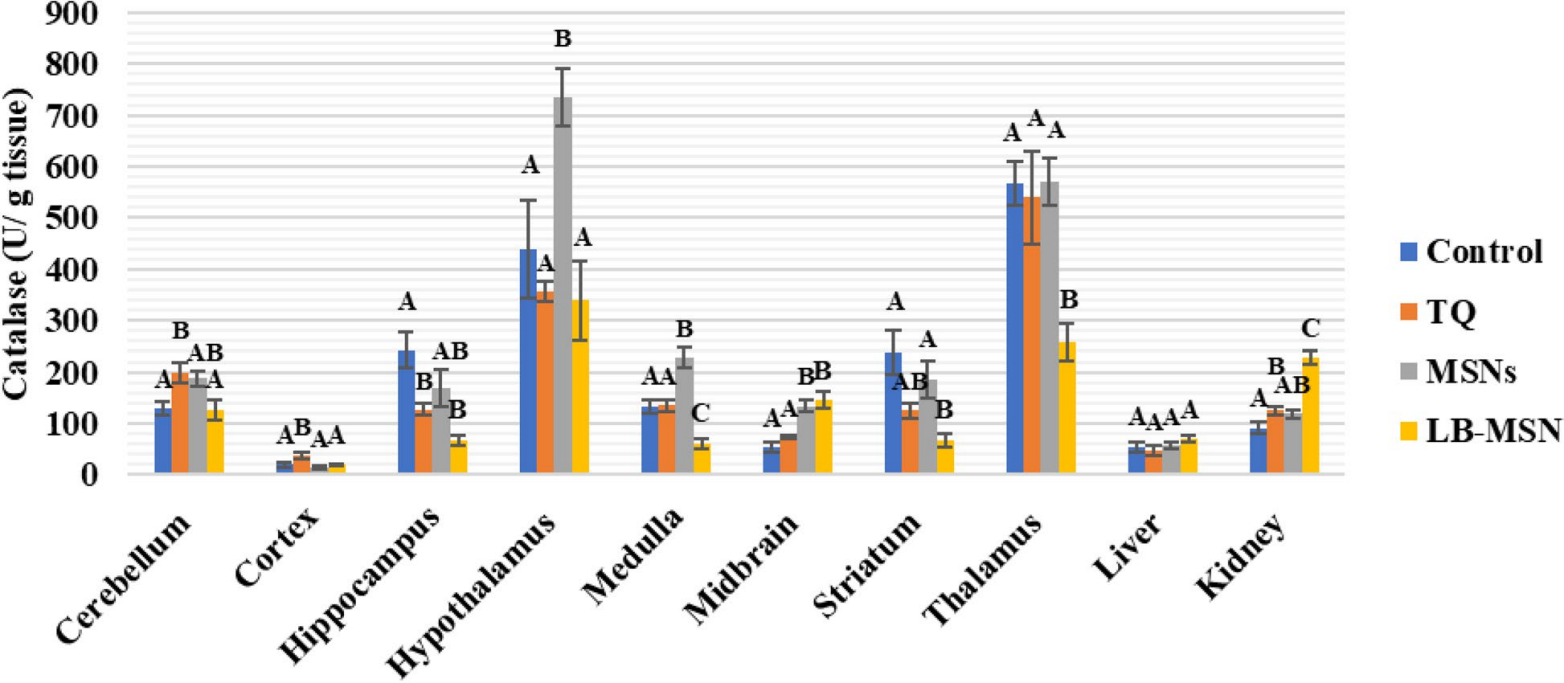
Catalase activity (U/g tissue) in the different areas after treatment with lipid bilayer-coated mesoporous silica nanoparticles loaded with thymoquinone

As can be seen in Fig. 6, except in the cerebellum (55.4%), brain (89.54%), kidney (37.19%), and hippocampus (37.19%), the catalase activity in the TQ group revealed no significant differences in all investigated areas compared with the negative control group values (−47.48%). Compared with the control values, catalase levels in the hypothalamus, medulla, and midbrain of MSNs exhibited considerable increases (105.68%, 67.93%, and 151.63%, respectively); however, no significant changes were noted in the remaining areas. The LB-MSNs group demonstrated significantly increased catalase activity in the midbrain and kidney than the negative control values (171.49% and 150.08%). Meanwhile, catalase levels in the hippocampus, medulla, striatum, and thalamus were significantly lower than in the control values (72.17%, 73.12%, 71.31%, and 54.53%, respectively) respectively).

#### Histopathological examination of the kidney, liver, and different brain areas

**Fig. 7:**
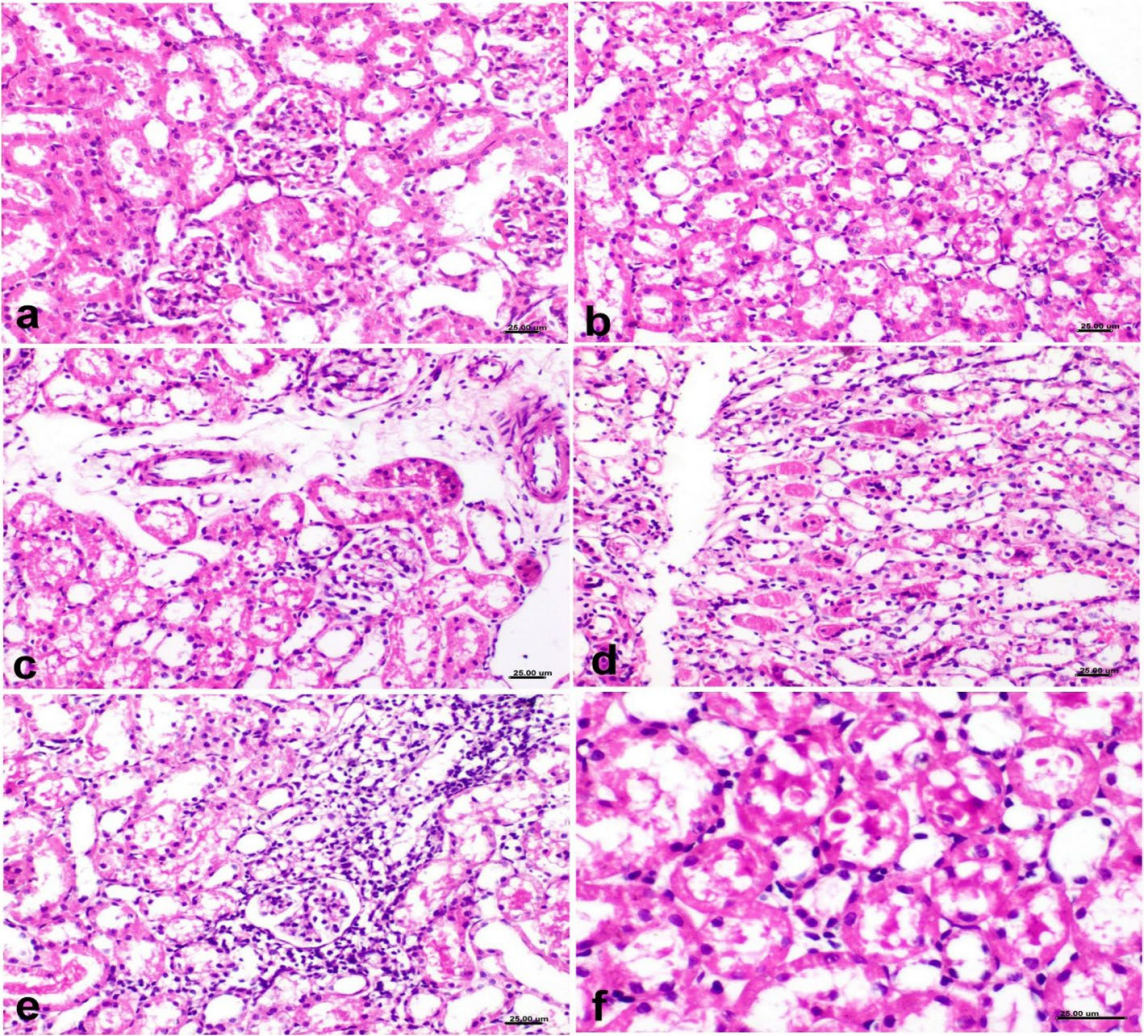
Hematoxylin and eosin-stained histological kidney sections (H & E x400). a) Control untreated rats showing a standard histological structure of the renal tubular epithelium, interstitial tissue, and glomeruli. b) TQ-treated rats showing vacuolization of the renal tubular epithelium that involves a few convoluted tubules in the renal cortex associated with few mononuclear cell infiltrations in the renal interstitial tissue. c) MSN-treated rats showing tubular epithelium vacuolization and necrosis with few mononuclear cell infiltration. d) MSN-treated rats showing cellular and hyaline aggregation in the tubular lumina. e) LB-MSN-treated rats showing necrobiotic and inflammatory reactions in the renal tissue. f) LB-MSN-treated rats showing renal tubular epithelium necrosis with hyaline droplet formation in the tubular epithelium

As seen in Fig. 7, according to microscopic testing (H & E x400), the control group had a standard histological structure of the kidney (Fig. 7a). The renal tissue of the TQ-treated group showed mild histopathological alterations; lesions were restricted to the renal cortex and included vacuolization of the renal tubular epithelium, especially the proximal convoluted tubules, with few mononuclear cell aggregations (Fig. 7b). Simultaneously, histological renal sections of the MSN-treated group had necrobiotic alterations in the renal tubular epithelium, which affected the proximal, middle, and distal tubules. Distal and collecting tubules in the renal cortex and medulla revealed lesions characterized by tubular epithelium degeneration and necrosis (Fig. 7c), cellular and proteinaceous cast aggregation, and interstitial mononuclear cell infiltration (Fig. 7d). Renal histopathological alterations in the LB-MSN-treated group were more severe than those in other groups; degenerative and necrotic reactions included the renal cortex and medulla and were associated with an inflammatory reaction involving the renal interstitium (Fig. 7e). The microscopic examination of different brain areas and liver revealed no difference between the treated and control groups.

## Discussion

The Blood-Brain Barrier guards the brain and prevents the entrance of foreign substances. Its function is based on the brain's robust endothelial barrier, which blocks the passage of materials [23]. Recently, scientists have revealed nanoparticle synthesis technology to overcome the action of BBB and succeed in delivering drugs to the brain. Nanoparticles are now considered excellent drug delivery systems, especially for neurological diseases. MSNs are one of the brain's most efficient drug delivery systems. Recently, MSNs revealed that therapeutic and diagnostic purposes support their application in biomedical research. The inner pores of MSNs can contain organic molecules with a high loading, such as fluorescent or MRI contrast agents. At the same time, the exterior surface can be selectively functionalized to provide a site-specific targeting capability, allowing for the realization of diverse intracellular delivery techniques. Pores can transport medicines or be employed to immobilize functioning enzymes [24]. MSNs can also be employed to immobilize functioning enzymes. These characteristics support the usage of MSNs *in vivo* in biomedical research rather than testing *in vitro* in outside cell experiments.

MSNs were confirmed to cross the BBB either by endocytosis or specific transporters [25]. They can cross the BBB according to physical and chemical properties, such as shape, zeta potential, and particle size [26]. The results of TEM imaging revealed that MSNs, MSN-TQ, and LB-MSNs were successfully prepared with spherical homogeneous morphology. Moreover, DLS confirmed the difference in the mean hydrodynamic diameters of the MSNs, MSN-TQ, and LB-MSNs, wherein LB-MSNs had the most significant value. The hydrodynamic diameter was due to the encapsulation of TQ to the MSNs with the addition of the lipid bilayer thickness. The PDI characterizes how the particles are uniformly and homogeneously distributed. The narrow range of the size distribution was measured as 0.1–0.5, and PDI values >0.5 referred to a wide range [27]. This study revealed a narrow distribution range for PDI with the following values: 0.234, 0.251, and 0.474 for MSNS, MSN-TQ, and LB-MSNs. This indicates the suitability of the prepared drug delivery systems with enhanced pharmacokinetic parameters (e.g., distribution and absorbance) [28]. This study's physical characterization tests demonstrated that the synthesized MSNs were optimal medication targeting and delivery systems to the brain. Due to their proper particle size, they stay in the bloodstream for a long time, evade the immune system, and cross the BBB, reaching various areas with different concentrations.

Zeta potential values supported physical characterization assessment and reported high stability of the prepared formulas; MSNs showed an average zeta value of −28.2 ± 2.48 mV and loading MSNs with TQ increased the formulation's stability demonstrated by a mean zeta value of −56.1 ± 6.74 mV. Coating with lipid bilayer slightly decreased the zeta value at −50.2 ± 6.35 mV, suggesting high stability. The negative charge of the zeta potential values was attributed to the encapsulated, negatively charged TQ molecules.

The release of TQ into the medium depends on the binding between TQ and the nanocarrier MSN, the permeability of the coat (LB), and the pH of the medium [12]. An *In vitro* release experiment of TQ from thymoquinone-loaded liposomes was described as negligible due to the lipophilicity of TQ [29]. As a result of including MSNs in our formulation, TQ's in vitro release profile suggested that the LB-MSNs formulation controlled the TQ release from the surface of the nanoparticles and internal pores, thereby making it a promising strategy to decide the desired TQ doses for different brain area s[30].

MDA, a well-known oxidative stress marker, is a byproduct of the peroxidation of polyunsaturated fatty acids in cells [31]. Lipid peroxidation, which has been linked to toxicity and is typically detected by MDA levels, is one of the most common symptoms of oxidative damage [27. NO is a neuromodulator agent that plays a vital role in brain neurotransmission. However, NO has shown conflicting roles in biological systems, as it is an oxidant at high levels and an antioxidant at low levels [29, 30]. GSH is a powerful endogenous antioxidant that regulates various metabolic activities [32]. GSH acts as an intracellular antioxidant defense against reactive oxygen species (ROS) [33]; it reacts directly with ROS in non-enzymatic reactions, resulting in ROS suppression [34]. Conversely, hydrogen peroxide (H_2_O_2_) is one of the effective ROS that elevates oxidative stress in the brain; the catalase enzyme is responsible for H_2_O_2_ decomposition [35].

TQ, the active ingredient of *Nigella sativa*, is known for its potent antioxidant activity. It reduces brain-induced oxidative stress [36]. Lipid-coated MSNs, which have the characteristics of MSNs and liposomes altogether, have been proven to cross the BBB [37–40]. The present study examines the distribution pattern of free TQ, TQ-loaded in LB-MSNs, and MSNs in the various brain areas and their subsequent effects on the oxidative and antioxidative stress biomarkers in different brain areas of the liver and kidney.

TQ inhibits NO production through inducible NO synthase suppression; TQ at 2, 5, or 10 mg/kg doses decreased NO and MDA metabolites in the hippocampus of the rat brain [37]. LB-MSNs have increased MDA and NO levels in the cerebellar tissue more than in the free TQ-treated and control groups. The increased MDA and NO levels are attributed to the lower TQ concentration reaching the cerebellar tissue than observed in the free TQ, as shown in Table 1. The control group had higher GSH levels in the cerebellum than the three treated groups (free TQ, MSN, and LB-MSNs), which follows the findings of Ferah et al. (2018), indicating the protective antioxidant defense effect of TQ against ROS that were generated in cerebellar tissues [41]. Furthermore, the catalase activity showed increased values in the free TQ and MSNs groups and decreased values in the LB-MSNs group, similar to the control group. The results indicate that the LB-MSNs coating succeeded in targeting TQ as an effective antioxidant to the cerebellum with a proper concentration, decreasing the ROS, specifically H_2_O_2_, the substrate for catalase reaction. This finding supports TQ's activity as a neurotoxicity inhibitor and preservative substance for cerebellar and cortex cells [30].

In the cortex, the NO level in the formulation group (LB-MSNs) was significantly lower than that in control, free TQ, and MSNs groups, whereas the MDA levels—an indicator of membrane lipid peroxidation—in the free TQ group were lower than those in the LB-MSNs group, which in turn was lower than the control group. The MDA and NO levels in the TQ group were consistent with the previous studies [37, 42, 43]. Although LB-MSNs significantly decreased the NO level, it showed a lower but nonsignificant MDA level. The GSH level was substantially increased in LB-MSNs, indicating TQ's adequate delivery and release. Regarding the catalase activity, its level increased only in the free TQ group compared with the control group. The lower catalase levels in LB-MSNs may be attributed to the controlled release of TQ in the cortical tissue.

In the hippocampus, the MDA and NO levels of the TQ group were lower than those of the control group, which is consistent with the results of [40, 44]; the LB-MSNs group showed even lower MDA and NO levels due to the facilitated delivery of TQ more to the hippocampal tissue, leading to its increased concentration. TQ competence was not limited to minimizing oxidative stress in the hippocampal area, but Merve et al. (2018) reported that TQ was efficient in increasing hippocampal neurons and limiting cell apoptosis by decreasing caspase-3 expression and PARP cleavage [45]. Catalase results supported the existence of TQ in hippocampus cells, where MSNs showed the lowest value for catalase among the four groups. Simultaneously, the GSH levels in the LB-MSNs group were higher but nonsignificant than those in the other groups.

Compared with the free TQ-treated group in the hypothalamus, the LB-MSN-treated group had increased MDA, and NO levels, which could result from MSNs alone since the formulation's difference in oxidative stress levels and MSNs groups was nonsignificant. Conversely, the GSH and catalase levels were similar in the free TQ and LB-MSN-treated groups, confirming that the TQ concentration reaching the hypothalamus may not be recognized.

In the medulla, compared with the control group, the LB-MSNs group demonstrated a substantial decrease in the MDA level and a significantly low NO level with lower GSH and catalase levels, suggesting the safe use of LB-MSNs as a drug targeting system for neurodegenerative diseases affecting the medullary tissue. This reach agrees with a previous study that proved that TQ efficiently counteracts the adverse effects of antioxidants in both the cortex and the medulla [46].

In the midbrain, the higher NO and MDA levels in the TQ and LB-MSN-treated groups nullified their use when targeting the midbrain and represented induced oxidative stress in the brain region. The results expressed in the TQ-treated group emphasized in this study are consistent with those of Sedaghat et al. [47]. Notably, encapsulating TQ in MSNs that boosted its transport to the midbrain may cause increased oxidative stress. However, catalase levels significantly increased in both MSNs and LB-MSN-treated groups. The increased catalase level may be considered proof of the protective characteristic of TQ as an antioxidant that prevents damage caused by high MDA and NO levels. This finding is consistent with that of Nasra et al. [48], who confirmed the efficiency of TQ in upregulating the catalase gene expression and supported the protective role of TQ.

Both NO and MDA levels showed nonsignificant differences among the four groups in the striatum. The results of our study disagree with those obtained by Ramachandran and Thangarajan [49], noting that in that study, researchers used a higher thymoquinone-loaded solid lipid nanoparticles (TQ-SLN) concentration (80 mg/kg) than that used in our study, which could be a possible explanation for these results. However, the same study agreed with the results of our study regarding the significantly elevated GSH level in the LB-MSNs group compared with that in the control group. The alleviated oxidative stress resulted in low catalase activity in both free TQ and LB-MSNs groups, with a lower value in the LB-MSNs group.

LB-MSNs have increased the MDA and NO levels in the thalamus more than in the free TQ and control groups. Nevertheless, the reverse occurred for GSH levels, wherein both MSNs and LB-MSN-treated groups showed decreased GSH values compared with the control group. The catalase value dramatically decreased only in the LB-MSN-treated group, which is justified by the controlled release of TQ. Unfortunately, the thalamus showed high ROS content with a negligible alleviating effect of the administered TQ doses.

The NO and MDA levels of TQ are consistent with previous studies [43, 44, 47]. In the liver tissue of the group that received the LB-MSNs formulation, there was a substantial reduction in NO levels compared with those in the control and TQ-treated groups; MDA levels of the LB-MSN-treated group were lower than those of the TQ group. Moreover, our formulation could keep the MDA level lower than the control's, demonstrating an antioxidant potency of the tested LB-MSNs formulation. In the kidneys, the formulation group had a greater NO level than the control and TQ groups, which applies that TQ effectively reverses renal tissue damage, revealing that TQ may be therapeutic to renal functioning during free radical therapy [46]. Additionally, the MDA level was higher than the control, although similar to the TQ group.

Our results showed that the LB-MSNs formulation successfully delivered TQ into the different brain areas, liver, and kidney. The TQ distribution data proved that TQ loading on MSNs altered the TQ distribution map throughout the brain, resulting in increased drug access, improved efficiency to the cortex and hippocampus, and better drug TQ delivery to the cerebellum hypothalamus, midbrain, and striatum. This allowed medications to be directed to specific areas of the brain.

It can be deduced that LB-MSNs could successfully decrease the oxidative stress induced in the brain, as indicated by the decrease in the NO and MDA levels in the cortex, hippocampus, medulla, and liver. The GSH levels are confirmed to increase in the cerebellum and cortex, along with the increased catalase in the cerebellum, cortex, and hippocampus.

Notably, MSNs caused a higher GSH concentration in the hypothalamus, striatum, cerebellum, and cortex, making them an excellent choice for our drug delivery system; however, this may need further investigation.

## Conclusion

LB-MSNs are capable nanoplatforms that can be used to target medications precisely to specific brain regions. The physical properties of the synthesized formulation were determined. It was established that they were suited for targeted therapy because their capacity to permeate the BBB and TQ was biodistributed over multiple brain regions. It was observed that different brain regions absorbed and reacted differently to TQ when embedded in LB-MSNs (as measured by oxidative stress markers). The results of this study may pave the way for the use of LB-MSNs as a practical method for targeting drugs to specific brain regions in several clinical and diagnostic applications.

## Data Availability

All data needed to support the conclusions are included in this article, and supplementary data are present in the supplemental materials. Additional data related to this paper can be requested from the author (hfahmy@sci.cu.edu.eg)

## Abbreviations

BBB: The Blood-Brain Barrier
TQ: Thymoquinone
MSNs: Mesoporous silica nanoparticles
LB-MSNs: Lipid bilayer-coated MSNs
FA: Folic acid
RGD: Arginine–glycine–aspartic acid
MS: Multiple sclerosis
CTAB: Cetyltrimethylammonium bromide
TEOS: Tetraethyl orthosilicate
DMSO: Dimethyl sulfoxide
PBS: Phosphate buffered saline
TEM: Transmission electron microscopy
PS: Particle size
DLS: Dynamic light scattering
ZP: Zeta potential
MDA: Malondialdehyde Lipid peroxidation
GSH: Reduced glutathione
NO: Nitric oxide
DHBS: 3, 5-dichloro-2-hydroxybenzene sulfonic acid
PDI: Polydispersity index
